# Effect of a moderate alcohol dose on physiological responses during rest and prolonged cycling

**DOI:** 10.1093/alcalc/agad079

**Published:** 2023-11-17

**Authors:** Andrew Marley, Marianna Bakali, Charlie Simpson

**Affiliations:** School of Applied Sciences, Division of Sport and Exercise Science, Abertay University, Dundee, United Kingdom; Faculty of Health and Life Sciences, Oxford Brookes University, Oxford, United Kingdom; Faculty of Health and Life Sciences, Oxford Brookes University, Oxford, United Kingdom

**Keywords:** ethanol, heart rate, submaximal cycling, oxidation, diuresis

## Abstract

Aim: We examined the acute effects of a moderate alcohol dose (48 g) ingested before prolonged cycling on acute physiological responses in eight healthy males (mean ± SD; 23 ± 2 years; 1.77 ± 0.04 m; 75.8 ± 4.1 kg). Methods: In a randomized order, euhydrated participants completed two experimental sessions with the sequence of 150-min seated at rest, 90-min of cycling at 50% of the maximal rate of oxygen consumption ($\dot{\textrm V}\textrm O$_2max_), 120-min seated at rest. Participants drank 250 mL of flavored squash with or without alcohol (vodka; ~16 g) at 10, 40, and 70 min of the initial resting phase, giving a cumulative fluid intake of 750 mL with 48 g of alcohol. Heart rate, blood glucose, breath alcohol concentration, and respiratory gasses were recorded throughout the entire trial with cumulative urine volume recorded during both rest phases. Results: Total carbohydrate (control = 115 ± 19 g: alcohol = 119 ± 21 g; *P* = 0.303) and lipid (control = 17 ± 4 g: alcohol = 20 ± 7 g; *P* = 0.169) oxidation was similar between conditions. Average heart rate was 7% higher in the alcohol condition (control = 111 ± 12 bpm; alcohol = 119 ± 11 bpm; *P* = 0.003). Blood glucose concentrations were similar between conditions during (*P* = 0.782) and after exercise (*P* = 0.247). Urine output was initially increased between conditions following alcohol ingestion before diminishing (*P* < 0.001) with no difference in total cumulative urine output (*P* = 0.331). Conclusion: Consuming an alcoholic drink containing 48 g of alcohol in the hour before moderate intensity sub-maximal aerobic exercise led to detectable increases in heart rate and rate of urine production with no effect on substrate use.

## Introduction

Alcohol is not commonly consumed prior to exercise due to its ergolytic properties (Vella and Cameron-Smith 2010) and obvious safety concerns. Anecdotal evidence suggests that some adults consume alcohol before cycling extended distances, either as part of a commute or as a social activity during rest stops in an extended recreational cycle. Nevertheless, there is limited research about the physiological effects of alcohol consumption before exercise.

Ethanol is the ingestible form of alcohol, contributing ~7 kcal·g^−1^ (Lands 1995). Once alcohol is ingested it is preferentially oxidized over other macronutrients (Schutz 2000). The primary site of alcohol metabolism is the liver with oxidation mainly completed by the alcohol dehydrogenase pathway with acetate as the end product (Cederbaum 2012). Most of this acetate is shuttled from the liver to peripheral tissues, such as muscle, to be converted to acetyl CoA and metabolized in the tricarboxylic acid cycle (Suter and Schutz 2008; Cederbaum 2012). The acetyl CoA derived from the shuttled acetate ‘replaces’ the acetyl CoA derived from fat to meet energy requirements (Cederbaum 2012). As such it has been suggested that this preferential use of acetyl CoA from alcohol oxidation to regenerate adenosine triphosphate lowers rates of fat oxidation in resting conditions (Suter 2000). With the theoretical maximal oxidation rate of alcohol being 0.1 g·kg^−1^·h^−1^ of lean body mass (Schutz 2000), it is unclear to what extent alcohol ingestion prior to exercise can alter exercising substrate use.

Few studies have investigated the acute effects of pre-exercise alcohol ingestion on exercise metabolism. Bond et al. (1984) used a crossover design with participants who were abstainers of alcohol or moderate consumers who ingested either 0 g·kg^−1^, 0.35 g·kg^−1^, and 0.69 g·kg^−1^ of a 95% ethanol solution, equating to either 0 g, 26 g, and 52 g of ethanol for a 75 kg individual, 30 min prior to completing a maximal graded exercise treadmill test until volitional exhaustion. There was no significant difference in maximal heart rate or maximum rate of oxygen consumption ($\dot{\textrm V}\textrm O$_2max_) between the three treatments, irrespective of whether participants were abstainers or habitual consumers of alcohol (Bond et al. 1984). Kendrick et al. (1993) also utilized a crossover design where four healthy males ingested ethanol (20 g) 10 min before and again 30 min into a 60-min run at 80–85% $\dot{\textrm V}\textrm O$_2max_. They observed a significant increase in heart rate by ~9%, with no difference in $\dot{\textrm V}\textrm O$_2_ during the run (Kendrick et al. 1993). More recently, thirteen trained male cyclists ingested 0.39 g·kg^−1^ fat free mass (FFM) ethanol, equivalent to ~22 g of alcohol per participant, or a volume matched water control before a self-paced 60-min time trial (Lecoultre and Schutz 2009). The authors reported a significantly increased exercising heart rate when normalized to power output, decreased blood glucose concentrations 40 min into the time trial and post-exercise, reductions in carbohydrate oxidation rates by 13% with no significant difference in lipid oxidation rates (Lecoultre and Schutz 2009). Conversely, in a well-controlled crossover study, seven recreationally active males remained seated at rest for 60 min and ingested ~6 g of alcohol as 0.1 g·kg^−1^·h^−1^ FFM of ethanol or a volume matched water control (Smith et al. 2021). Next, participants cycled at 55% $\dot{\textrm V}\textrm O$_2max_ for 120 min (Smith et al. 2021). The low dose of alcohol administered was chosen to avoid excess alcohol greater than the theoretical maximal oxidizing rate to spill into systemic circulation, preventing an accumulation of blood alcohol. There was no significant difference between conditions in muscle glycogen utilization, carbohydrate oxidation, lipid oxidation, plasma glucose concentration, or plasma nonesterified fatty acid concentrations (Smith et al. 2021). The only significant difference was a 19% increase in blood lactate concentration following alcohol ingestion in the first 30 min of the 60 min rest period prior to exercise (Smith et al. 2021). Therefore, conflicting evidence exists as to the effect of alcohol consumption on exercise metabolism with larger doses potentially needed to alter substrate use.

Alcohol exerts a diuretic effect via inhibited vasopressin release which increases urine output due to increased renal water excretion (Harper et al. 2018). Vasopressin is released by the posterior pituitary gland and decreases water excretion in the kidneys by increasing expression of aquaporin-2 channels into the apical membrane of tubular epithelial cells (Boone and Deen 2008). Exercise increases vasopressin excretion to allow enhanced water availability for metabolic processes and sweating (Hew-Butler 2010). The competing actions of exercise and alcohol consumption on vasopressin release makes it unclear if alcohol consumption prior to exercises influences urine output. Of the limited research in this field, Castro-Sepulveda et al. (2016) recruited seven male professional soccer players who consumed either 0.7 L of alcoholic beer (4.6% alcohol by volume (ABV); 25 g alcohol), non-alcoholic beer, or water. Participants then completed 45 min of steady state treadmill running at 65% HR_max_ with no difference in urine output recorded throughout (Castro-Sepulveda et al. 2016). The diuretic effect of alcohol is blunted if the body is hypohydrated (Hobson and Maughan 2010). It is unclear if moderate alcohol ingestion prior to engaging in prolonged submaximal exercise alters urine output.

The aim of this study is to evaluate the effect of a moderate dose of alcohol on respiratory, cardiorespiratory, and urinary responses to 90 min of cycling at 50% $\dot{\textrm V}\textrm O$_2max_. We hypothesize that ingestion of 48 g of alcohol will cause a decrease in the absolute rates of carbohydrate and lipid oxidation with an increase in rate of urine production and cumulative urine output with an increase in exercising heart rate.

## Materials and Methods

### Participants

Eight recreationally active males who were habitual drinkers participated (mean ± SD; age: 23 ± 2 years; height: 1.77 ± 0.04 m; weight: 75.8 ± 4.1 kg). Exclusion criteria included smokers, abstainers of alcohol, reported alcoholism, and underlying health conditions which may influence fluid balance and respiratory data. This study was approved by the ethics committee of Oxford Brookes University with all participants providing written informed consent and free to withdraw at any time.

### Experimental design

Participants completed two trials (control and alcohol) in a randomized crossover design. We did not attempt to blind subjects to treatment because there is no suitable placebo that can mask the effects of a moderate dose of alcohol. Each participant visited the laboratory on three occasions separated by a minimum of 5 days.

### $\dot{\textrm V}\textrm O$ _2max_ testing

At visit one, participants weight and height was measured using calibrated electronic scales and a stadiometer before being fitted with a heart rate monitor (Polar FT31, Kempele, Finland). After individually adjusting the seat height on the electronically braked ergometer (Lode Corival, Groningen, Netherlands) participants then self-selected the starting resistance to be between 50 and 100 W. Participants then completed 4-min stages, maintaining a cadence of 60RPM with expired gas collected in the final 60s of each stage using PVC collection bags (Cranlea, UK). The O_2_ and CO_2_ fractions were measured (Servomex 5200, Egham, Surrey, United Kingdom) prior to determination of gas volume (Dry Gas Meter, Harvard Apparatus, Holliston, USA). Intensity was increased by 30 W per stage until volitional exhaustion with $\dot{\textrm V}\textrm O$_2max_ and W_max_ recorded.

### Experimental trial standardization

Participants were asked to record and then replicate their self-selected food and drink consumption in the 24 h before the experimental trials. Participants were asked to avoid consuming alcohol or caffeine for 24 h before trials and to limit strenuous exercise for 48 h before trials. Compliance was assessed verbally before each trial. Participants consumed a standardized breakfast 2 h before experimental trials which included 500 mL water, 600 mL orange juice (Tesco Smooth Orange Juice) and two 37 g cereal bars (Nutrigrain). The breakfast provided the following nutritional intake; kcal: 515; fat: 7 g; carbohydrate: 106 g; protein: 7 g; ~total fluid: 1112 mL.

### Drink composition

Participants consumed three boluses of 250 mL as 250 mL of orange squash (control; Robinsons Orange Squash-No Added Sugar) or 200 mL of orange squash with 50 mL of vodka (alcohol; 40% ABV, 16 g ethanol, Smirnoff).

### Experimental trials

**Figure 1:**
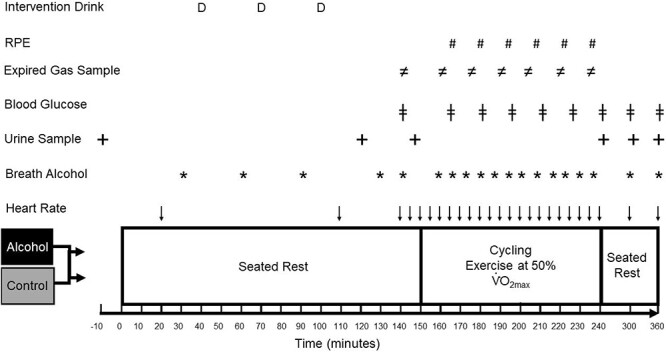
Schematic overview of experimental timeline. $\dot{\textrm V}\textrm O$_2max_ = maximal oxygen uptake

For a schematic overview see Fig. 1. Upon arrival participants were asked to provide a urine sample with volume and urine osmolality assessed (Osmocheck; Vitech Scientific, Horsham, UK). Body mass was then recorded before baseline breath alcohol concentrations were recorded (Dräger Alcotest 5510, Drägerwerk AG & Co. KGaA, Lübeck, Germany). Pre-exercise measures were conducted between 0 and 150 min into the trial with the participant remaining seated throughout. Heart rate, breath alcohol, blood glucose (Ascensia Contour Blood Glucose Meter) and urine volume were recorded with a 5 min expired air sample collected and analyzed (Fig. 1). The test drinks were consumed at 40, 70, and 100 min into trials. After 150 min of rest participants completed 90 min of steady state cycling exercise at a pace of ~50% of individual’s $\dot{\textrm V}\textrm O$_2max_. During exercise, heart rate was recorded every 5 min, rate of perceived exertion (RPE) every 14 min, breath alcohol every 7 min, blood glucose every 15 min with a 60s expired air sample collected every 15 min (Fig. 1). All participants consumed 200 mL of water at 165, 195, 225, and 240 min (total fluid intake of trial 1550 mL). After cycling, participants remained seated for a further 120 min with heart rate, blood glucose, and urine volume recorded (Fig. 1).

Substrate oxidation rates for carbohydrate and lipid were calculated by using the equations of Jeukendrup and Wallis (2005) from the observed $\dot{\textrm V}\textrm O$_2_ and V̇CO_2_, assuming negligible contribution from protein oxidation. Alcohol oxidation was assumed to be complete at the maximal hepatic oxidation rate reported by Schutz (2000). Measured respiratory exchange ratio (RER) was adjusted for alcohol oxidation by subtracting $\dot{\textrm V}\textrm O$_2_ and V̇CO_2_ associated with the complete oxidation of alcohol of 3O_2_ and 2CO_2_ per mol of alcohol from measured $\dot{\textrm V}\textrm O$_2_ and V̇CO_2_.

### Statistical analysis

Data were assessed for normality using the Shapiro–Wilk test. Paired sample t-tests were performed on initial measures of urine volume and osmolality to ascertain if participants completed both experimental trials in a similar state of hydration. All data were analyzed via repeated measures ANOVA (The Jamovi Project, version 2.2.1) with LSD post hocs used if significance was detected. All data is presented as means ± SD with significance set at *P* < 0.05.

## Results

Body mass was similar between conditions at baseline (control = 75.8 ± 4.7 kg; alcohol = 75.6 ± 4.7 kg; *P* = 0.925) and at the end of trials (control = 74.7 ± 4.7 kg; alcohol = 74.4 ± 4.5 kg; *P* = 0.893). Body mass decreased after both trials (*P* < 0.001) with no significant difference in body mass loss between conditions (*P* = 0.909).

**Figure 2:**
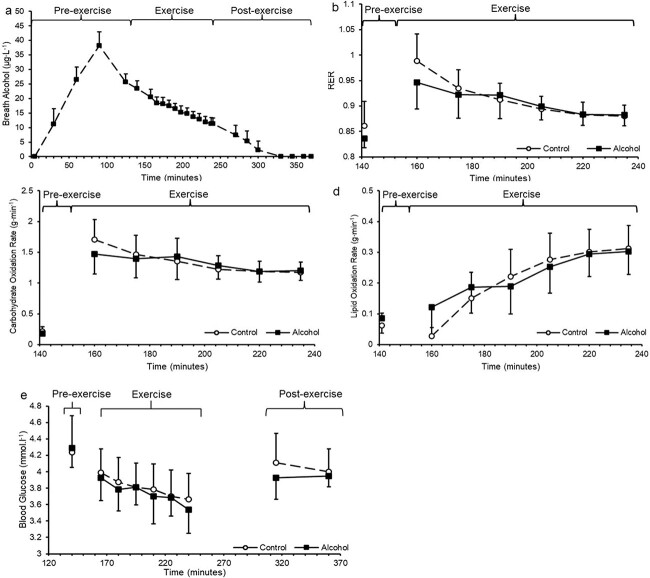
Respiratory and substrate oxidation data. *n* = 8 with all data presented as mean ± SD. (a) RER in control and alcohol conditions. (b) Carbohydrate oxidation rates in control and alcohol conditions. (c) Lipid oxidation rates in control and alcohol conditions. (d) Breath alcohol concentrations in alcohol condition. (e) Blood glucose (mmol·L^−1^) in control and alcohol conditions at pre, during and post exercise

Alcohol consumption increased breath alcohol concentrations (*P* < 0.001), peaking at 38 ± 5 μg·L^–1^ after 90 min of the seated rest before decreasing as the trial progressed (Fig. 2a).

After drink consumption during seated rest, RER was similar between conditions (*P* = 0.560; Fig. 2b). During exercise RER decreased in both conditions as exercise progressed (*P* < 0.001) with no difference between conditions (*P* = 0.132; Fig. 2b). Carbohydrate oxidation rates were similar between conditions during seated rest after drink consumption (control = 0.23 ± 0.08 g·min^−1^; alcohol = 0.18 ± 0.05 g·min^−1^; *P* = 0.077; Fig. 2c). During exercise, carbohydrate oxidation rates decreased as exercise progressed in both conditions (*P* < 0.001) with no difference between conditions (*P* = 0.056; Fig. 2c). Total carbohydrate oxidized during exercise in the control condition was 115 ± 19 g and in the alcohol condition was 119 ± 21 g (*P* = 0.303). Lipid oxidation rates were similar between conditions during seated rest after drink consumption (control = 0.06 ± 0.03 g·min^−1^; alcohol = 0.09 ± 0.02 g·min^−1^; *P* = 0.114; Fig. 2d). During exercise, lipid oxidation rates increased in both conditions as exercise progressed (*P* < 0.001) with no difference between conditions (*P* = 0.113; Fig. 2d). Total lipid oxidized during exercise in the control condition was 17 ± 4 g and in the alcohol condition was 20 ± 7 g (*P* = 0.169).

Blood glucose concentrations were similar between conditions after drink consumption during seated rest (control = 4.2 ± 0.3 mmol·L^−1^; alcohol = 4.3 ± 0.2 mmol·L^−1^; *P* = 0.727; Fig. 2e). Blood glucose decreased in both groups as exercise progressed (*P* < 0.001) with no difference between conditions (*P* = 0.782; Fig. 2e). After exercise, blood glucose remained stable (*P* = 0.454) with no difference between groups (*P* = 0.247; Fig. 2e).

**Figure 3:**
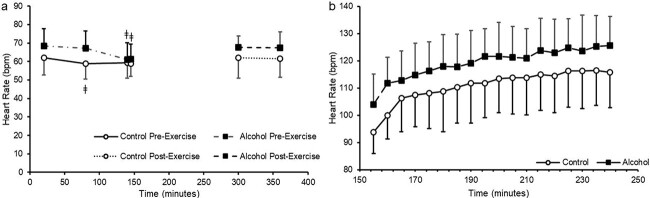
Heart rate during experimental trial. *n* = 8 with all data presented as mean ± SD. (a) Pre and post-exercise heart rate. (b) Heart rate during exercise. ǂ = significant within group difference from first recorded heart rate pre-exercise

Alcohol consumption significantly increased heart rate during seated rest before exercise (*P* = 0.004; Fig. 3a). Heart rate significantly increased in both conditions as exercise progressed (*P* < 0.001) with no difference in rate of increase between conditions (*P* = 0.935; Fig. 3b). Average heart rate during exercise was 7% greater in the alcohol condition (control = 111 ± 12 bpm; alcohol = 119 ± 11 bpm; *P* = 0.003). After exercise, heart rate remained stable (*P* = 0.705) with no difference between conditions (*P* = 0.820; Fig. 3a). RPE during exercise increased in both conditions as exercise progressed (*P* < 0.001) with no difference between conditions (*P* = 0.780).

**Figure 4:**
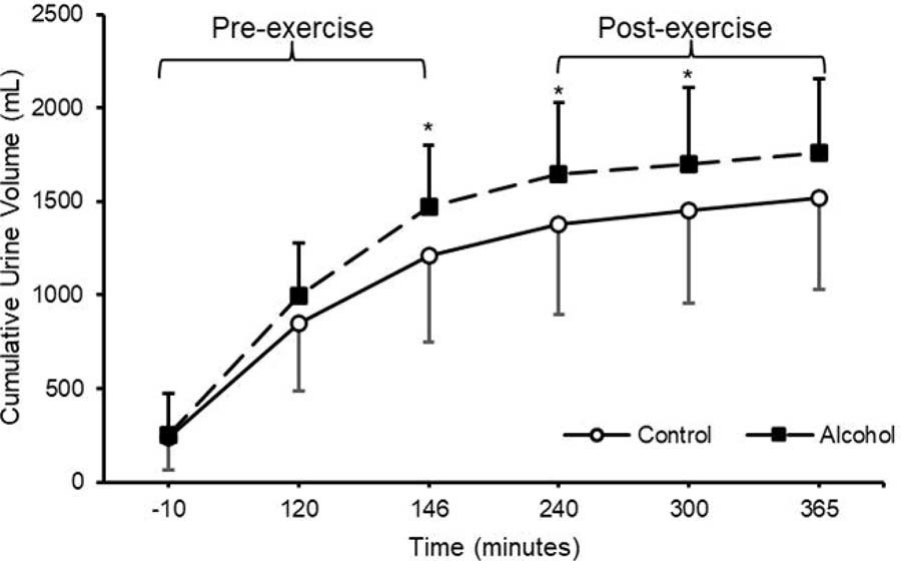
Cumulative urine output in control and alcohol conditions. *n* = 8 with all data presented as mean ± SD * = significant difference between groups at same time point

Upon arrival for the experimental trials participants presented with similar urine volumes (control = 237 ± 171 mL; alcohol = 253 ± 218 mL; *P* = 0.844; Fig. 4) and urine osmolality (control = 394 ± 189 mOsm; alcohol = 535 ± 369 mOsm; *P* = 0.253). Cumulative urine output increased in both conditions (*P* < 0.001) with an accelerated increase in the alcohol condition (*P* < 0.001; Fig. 4) However, final cumulative urine output was not significantly different between conditions (baseline *P* = 0.881; 120 min *P* = 0.367; 146 min *P* = 0.022, 240 min *P* < 0.001, 300 min *P* < 0.001, 365 min *P* = 0.311; Fig. 4).

## Discussion

The purpose of this study was to ascertain if ingesting 48 g of alcohol prior to 90-min of steady state cycling exercise at 50% $\dot{\textrm V}\textrm O$_2max_ would significantly decrease absolute rate of carbohydrate and lipid oxidation during exercise and accelerate urine production. Total carbohydrate and lipid oxidized were not different between conditions (Fig. 2c and d) with similar blood glucose concentrations during exercise (Fig. 2e). Alcohol consumption significantly increased average heart rate during exercise however was similar to the control condition following exercise (Fig. 3a and b) with RPE unchanged between conditions. Alcohol consumption accelerated the initial rate of urine output; however, this increase was transient with final cumulative urine output similar between conditions (Fig. 4). As such, moderate alcohol ingestion (48 g) prior to undertaking prolonged steady state cycling for 90 min does not significantly influence substrate oxidation during exercise with an increase in exercising heart rate and an initial increase in urine output.

There was no difference in carbohydrate or lipid oxidation rates with total oxidized substrate similar between conditions during 90 min of cycling exercise at 50% $\dot{\textrm V}\textrm O$_2max_ after ingesting 48 g of alcohol (150 mL of 40% ABV vodka) (Fig. 2c and d). Smith et al. (2021) found that ingesting a quantity of alcohol to match the theoretical maximal oxidation rate of ethanol of 0.1 g·kg^−1^FFM·h^−1^ (~20 mL of 40% ABV vodka; ~6 g ethanol) before cycling for 120 min at 55% $\dot{\textrm V}\textrm O$_2max_ did not significantly alter substrate use over a volume matched water control. Smith et al. (2021) suggested that a greater quantity of alcohol consumed may have supressed hepatic glucose production and reduced glucose uptake in to skeletal muscle, glycogen utilization and/or fatty acid utilization (Jorfeldt and Juhlin-Dannfelt 1978; Smith et al. 2021). However, in our study carbohydrate and lipid oxidation rates between conditions were similar (Fig. 2c and d), supporting the work of Smith et al. (2021). It is possible that ingesting moderate quantities of alcohol in the present study (48 g), a greater quantity than that of Smith *et al.* (~6 g of alcohol), which matched the theoretical maximal oxidizing rate of ethanol, does not significantly alter substrate oxidation during exercise due to the relatively small energy contribution from oxidizing alcohol at a maximal rate of 0.1 g·kg^−1^FFM·h^−1^. As such, the hypothesis that ingesting 48 g of alcohol prior to prolonged cycling exercise would reduce total carbohydrate and lipid which is oxidized is rejected.

There were no significant differences in blood glucose concentrations between the conditions (Fig. 2e). A review by Steiner et al. (2015) concluded that alcohol ingestion can either have no effect or reduce blood glucose concentrations in humans. The varying outcomes observed in different studies may be attributed to the differing nutritional statuses of the participants. Participants were asked to replicate their dietary food intake in the 24 h prior to attending the laboratory and consumed the same standardized breakfast with no significant differences in blood glucose concentrations between treatment conditions (Fig. 2e). This also substantiates the work of Smith et al. (2021) who did not observe any differences in blood glucose concentration following alcohol ingestion. Previously, alcohol ingestion prior to completing a 60-min cycling time trial in trained endurance cyclists decreased blood glucose concentrations (Lecoultre and Schutz 2009). It has been suggested that alcohol acutely inhibits hepatic glucose production via reductions in gluconeogenesis (Siler et al. 1998; Steiner et al. 2015). Intense and prolonged exercise requires elevated carbohydrate oxidation within skeletal muscle with increased hepatic glycogenolysis and gluconeogenesis supplying the additional required glucose (van Loon et al. 2001; Trefts et al. 2015). Therefore, alcohol consumption prior to prolonged intense exercise may decrease blood glucose concentrations due to increased carbohydrate uptake and utilization in skeletal muscle with inhibited hepatic glucose production. Graham (1983) reported that alcohol ingestion of 2.5 mL·kg^−1^ of 40%ABV vodka, equating to ~46 g of alcohol, did not significantly influence blood glucose levels from a volume matched water control during intermittent cycling exercise at 45% $\dot{\textrm V}\textrm O$_2max_ during the first 90 min of a 180 min protocol with reductions in blood glucose concentration following alcohol ingestion only present in the latter half of the exercise. Therefore, it is possible that the exercise protocol in the present study lacked sufficient duration to significantly alter blood glucose concentrations with available muscle glycogen and blood glucose able to supply the exercising muscle with substrate. In the present study, alcohol (48 g) consumed prior to 90 min of low intensity exercise does not significantly change blood glucose concentrations, possibly due to the lower carbohydrate requirements of skeletal muscle when compared to intense exercise.

Average heart rate during exercise was significantly higher following alcohol consumption (Fig. 3b). Other researchers have reported that heart rate during submaximal exercise is increased following a similar quantity of alcohol ingestion (Kendrick et al. 1993; Ferreira et al. 2004). Alcohol consumption elicits a vasodilatory effect, decreasing peripheral resistance which increases cutaneous blood flow (Daanen 2003). It has been suggested that this increased cutaneous blood flow results in an elevated heart rate during work matched exercise to sufficiently supply the muscles with blood (Blomqvist et al. 1970; Lecoultre and Schutz 2009). As alcohol ingestion prior to cycling ergometer exercise does not influence the catecholamine response to exercise, it is unlikely that the observed increase in heart rate in our study is due to changes in circulating catecholamine concentrations (Heikkonen et al. 1991). Acute alcohol consumption of varying doses also decreases blood pressure (Tasnim et al. 2020). An acute decrease in blood pressure, coupled with increased cutaneous blood flow, may lead to an increase in heart rate to increase cardiac output to effectively maintain blood supply to exercising muscle. However, future research where blood pressure and peripheral blood flow is monitored, is required to elucidate if that occurs.

Alcohol consumption accelerated cumulative urine output with no difference in final total produced urine and no difference between conditions for urine osmolality (Fig. 4). The American College of Sports Medicine (2007) state that urine osmolality ≤700 mOsm·kg^−1^ is indicative of euhydration. As there were no significant differences in body mass, urine volume, or urine osmolality upon arrival to the laboratory with all participants presenting a urine osmolality of ≤700 mOsm in both conditions, it can be assumed that all participants were euhydrated. Previously, 20 older males completed a randomized crossover trial and consumed beer, wine, or spirits, providing ~30 g of alcohol each, and volume matched non-alcoholic options. Only alcoholic wine and spirits provided a small and transient diuretic effect, increasing urine output in the 4 h after consumption but not increasing total cumulative urine output in the 24 h after consumption (Polhuis et al. 2017). As such the presented results corroborate these findings in a young male cohort with alcohol inducing a transient diuretic effect. Alcohol and water consumption decreases vasopressin secretion, with alcohol suppressing vasopressin more effectively (Sailer et al. 2020). This may explain why cumulative urine output was significantly greater at 146–300 min into the trial (Fig. 4).

In the present study, participants were administered with six standard UK measures of alcohol, which was sufficient to elicit participants to the breath alcohol drink drive limit in England of 35 μg·mL^−1^ (UK Government 2022; Fig. 2a). Most research which evaluates the effect of alcohol on exercise normalizes ethanol ingestion to participants FFM (Lecoultre and Schutz 2009; Smith et al. 2021). However, as this does not follow traditional drinking practices, we decided to provide standard drink measures. It is possible that normalizing ethanol consumption to participants FFM before steady state submaximal exercise may have altered substrate use. Future work should seek to provide a more complete overview of fluid balance by also monitoring perspiration rates and electrolyte concentrations of the urine following alcohol ingestion and steady state submaximal exercise.

Ingestion of 48 g of alcohol does not significantly alter carbohydrate oxidation, lipid oxidation, or blood glucose concentrations when compared to a volume matched water control during steady state submaximal cycling exercise. Cumulative urine output was accelerated following alcohol consumption with higher average heart rate throughout submaximal exercise. This has implications for cycling intensity monitoring for cyclists who engage in alcohol consumption as part of a recreational stop.

## Data Availability

Data is available upon reasonable request.
